# Phosphodiesterase 10A Inhibitor Modulates Right Ventricular Outflow Tract Electrophysiological Activities and Calcium Homeostasis via the cGMP/PKG Pathway

**DOI:** 10.1111/jcmm.70480

**Published:** 2025-03-11

**Authors:** Feng‐Zhi Lin, Yao‐Chang Chen, Hsiang‐Yu Yang, Wei‐Shiang Lin, Yen‐Yu Lu

**Affiliations:** ^1^ Department of Biomedical Engineering and Institute of Physiology National Defense Medical Center Taipei Taiwan; ^2^ Grade Institute of Life Sciences National Defense Medical Center Taipei Taiwan; ^3^ Department of Biochemistry National Defense Medical Center Taipei Taiwan; ^4^ Division of Cardiovascular Surgery, Department of Surgery, Tri‐Service General Hospital, National Defense Medical Center Taipei Taiwan; ^5^ Division of Cardiology, Department of Internal Medicine, Tri‐Service General Hospital National Defense Medical Center Taipei Taiwan; ^6^ Fu Jen Catholic University School of Medicine New Taipei City Taiwan; ^7^ Division of Cardiology, Department of Internal Medicine Sijhih Cathay General Hospital New Taipei City Taiwan

**Keywords:** Ca^2+^ regulation, phosphodiesterase 10A inhibitor, right ventricular outflow tract

## Abstract

Phosphodiesterase inhibitors regulate intracellular Ca^2+^ of cardiomyocytes through enhancing second messenger signalling. This study aimed to investigate whether TP‐10, a selective phosphodiesterase10A inhibitor, modulates Ca^2+^ cycling, attenuating arrhythmogenesis in the right ventricular outflow tract (RVOT). Right ventricular tissues from New Zealand white rabbits were harvested, and electromechanical analyses of ventricular tissues were conducted. Intracellular Ca^2+^ was monitored using Fluo‐3, and ionic current was recorded using patch‐clamp in isolated cardiomyocytes. Tissues from RVOT exhibited a reduction in action potential duration at both 50% and 90% repolarisation following treatment with TP‐10. This treatment also inhibited burst firing induced by isoproterenol (ISO) in RVOT tissues, an effect that was nullified by thapsigargin. The protein kinase G inhibitor KT5823, whether used alone or in conjunction with TP‐10, also suppressed ISO‐induced burst firing in these tissues. Compared to the control group, RVOT cardiomyocytes treated with TP‐10 demonstrated enhanced amplitudes of Ca^2+^ transients and increased stores of Ca^2+^ in the sarcoplasmic reticulum. Although the L‐type Ca^2+^ current was diminished in TP‐10‐treated cardiomyocytes, the current from the Na^+^‐Ca^2+^ exchanger was elevated. Furthermore, the density of late Na^+^ current was significantly reduced in these treated cardiomyocytes. TP‐10 administration also resulted in increased levels of calcium regulatory proteins, specifically phosphorylated phospholamban at Thr17 and sarcoplasmic/endoplasmic reticulum Ca^2+^ ATPase 2a. Our findings indicate that TP‐10 attenuates ISO‐induced arrhythmic events in RVOT tissues via cGMP‐mediated modulation of intracellular Ca^2+^ regulation.

## 1 Introduction

Acting as second messengers, cyclic adenosine monophosphate (cAMP) and cyclic guanosine monophosphate (cGMP) regulate a variety of cardiac functions, including inotropic and chronotropic activities, while also playing a role in structural remodelling [1]. Dysregulated cAMP/cGMP signalling, due to impaired synthesis and breakdown, leads to intracellular Ca^2+^ imbalance, cardiac dysfunction and arrhythmias, contributing to the mechanisms of arrhythmogenesis and heart failure [2, 3]. Phosphodiesterases (PDEs), which include the PDE1 to PDE11 families, specifically degrade cAMP and/or cGMP, thus playing unique roles in different cardiac cell types and influencing various cardiac pathologies [4, 5]. In a therapeutic context, PDE inhibitors can be utilised to manage heart failure (HF) by enhancing the signalling pathways mediated by cAMP or cGMP, potentially leading to improvements in intracellular Ca^2+^ cycling [6].

PDE10A was first recognised as a dual cAMP/cGMP phosphodiesterase capable of hydrolysing both molecules [7]. Its expression in neurons within the human striatum has led to the development of various PDE10A inhibitors intended for the treatment of psychiatric and neurodegenerative disorders [8]. Although the specific role of PDE10A in the cardiovascular system is not yet fully understood, studies have indicated that its expression is significantly increased in the failing hearts of both mice and humans [9]. Given its function in regulating intracellular levels of cAMP and cGMP, inhibiting PDE10A may enhance cardiac function, positioning it as a promising therapeutic approach for managing HF and cardiac arrhythmias [10]. Importantly, several PDE10A inhibitors have undergone testing in human clinical trials, suggesting that PDE10A could serve as a safe and viable therapeutic target [11, 12, 13]. Research suggests that PDE10A inhibition could also play a protective role in heart disease by influencing cardiomyocyte survival under stress conditions [10]. Consequently, TP‐10, a selective PDE10A inhibitor, may hold significant therapeutic potential for various cardiovascular conditions. However, the application of TP‐10 in the cardiovascular system remains largely uncharacterised and requires further investigation.

Arrhythmogenesis in the right ventricular outflow tract (RVOT) holds significant clinical importance due to its potential to induce serious arrhythmias that adversely affect patients' quality of life, necessitating careful management and targeted treatment strategies [14]. The RVOT is a primary site for the emergence of premature ventricular contractions and idiopathic ventricular tachyarrhythmias, in addition to being associated with long QT syndrome and Brugada syndrome [15]. Triggered electrical activity arising from the RVOT plays a crucial role in the onset of life‐threatening ventricular fibrillation, highlighting the importance of ablation techniques to remove these triggers and reduce the risk of malignant arrhythmias [16]. Research into RVOT arrhythmogenesis is essential for advancing diagnosis and treatment, thereby improving patient outcomes. Myocytes from the RVOT of rabbits, which exhibit unique electrophysiological characteristics and increased susceptibility to arrhythmogenesis, are commonly used in studies of RVOT arrhythmogenesis [16].

The aim of this study was to assess whether TP‐10 reduces isoproterenol (ISO)‐induced ventricular arrhythmias in the RVOT and to elucidate the underlying mechanisms involved.

## 2 Methods

### 2.1 Preparing Ventricular Tissues for Electromechanical Assessments

All animal experiments received approval from the Institutional Animal Care and Use Committee at the National Defense Medical Center in Taipei, Taiwan (IACUC‐22‐222) and were conducted in compliance with the National Institutes of Health's *Guide for the Care and Use of Laboratory Animals* (*the Guide*).

In this investigation, male New Zealand white rabbits, each weighing between 2 and 3 kg and aged 6–8 months, were employed. The rabbit heart is frequently used as a model in electrophysiological studies due to several similarities in electrophysiological properties, including action potential characteristics, Ca^2+^ handling mechanisms, and ion channel expression and function, when compared to the human heart, particularly in contrast to rodent models. These similarities are essential for research on arrhythmias, cardiac function and responses to drugs [17, 18]. The rabbits were maintained in stainless steel cages within a controlled environment, where the temperature was kept between 20°C and 22°C and humidity levels ranged from 50% to 70%. They were subjected to a 12‐h light/dark cycle and were provided with unrestricted access to standard food and deionised water. The echocardiography data of the rabbits were recorded (Figure S1) to demonstrate their healthy status.

For anaesthesia, the rabbits were given an intramuscular injection of a combination of zoletil 50 (10 mg/kg) and xylazine (5 mg/kg), followed by the inhalation of an overdose of isoflurane (5% in oxygen) delivered through a vaporiser. Euthanasia was subsequently conducted according to previously described methods [19]. The effectiveness of the anaesthetic was verified by the absence of motor responses as well as corneal reflexes in response to pain stimuli. After administering heparin (1000 units/kg), the hearts were excised via sternotomy in accordance with previously established protocols [19].

**FIGURE 1:**
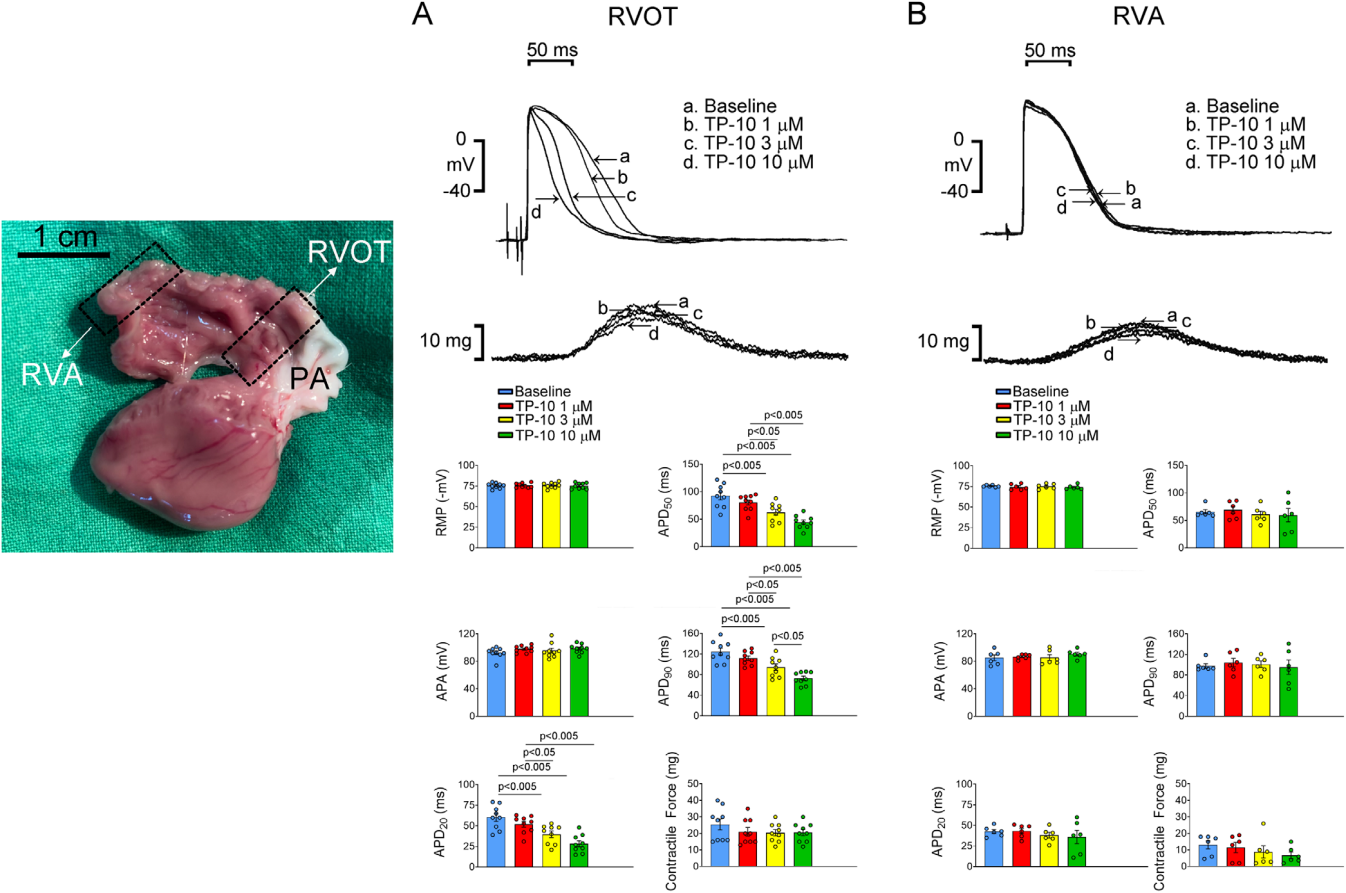
Effects of TP‐10 on action potentials of the RVA and RVOT tissues. (A) (Upper) Representative action potentials and contractile forces from the RVA tissues treated with TP‐10, and (below) the mean values (*N* = 9). (B) (Upper) Representative action potentials and contractile forces from RVOT tissues treated with TP‐10, and (below) the mean values (*N* = 6). APA, action potential amplitude; RMP, resting membrane potential; RVA, right ventricular apex; RVOT, right ventricular outflow tract; APD_20_, APD_50_, and APD_90_, action potential duration at repolarisation levels of 20%, 50%, and 90% of the action potential amplitude, respectively.

Following euthanasia, tissues from the RVOT and the right ventricular apex (RVA) were excised from rabbits (Figure 1) and anchored to a tissue bath for subsequent analysis based on established protocols [20]. The preparations were continuously superfused with Tyrode's solution at a flow rate of 3 mL/min and aerated with a gas mixture comprising 95% O_2_ and 5% CO_2_ at a maintained temperature of 37°C. An equilibration period of one hour was allowed before conducting electrophysiological evaluations.

Action potentials (APs) of the RV tissues were measured using a borosilicate glass micropipette filled with 3 M KCl, connected to an electrometer (WPI Duo 773, World Precision Instruments, USA), in accordance with established protocols [21]. The resulting signals were digitally acquired via a data acquisition system featuring a cutoff frequency of 10 kHz, processed through a low‐pass filter, and recorded with 16‐bit precision at a sampling rate of 125 kHz. A pulse stimulation lasting 1 ms was produced using a stimulator as previously described cols [21].

Action potential durations (APDs) in the right ventricular (RV) preparations were measured under 2 Hz pulse stimulation. The action potential amplitude (APA) was determined as the difference between the peak depolarisation potential and the resting membrane potential (RMP). APD at 20%, 50% and 90% repolarisation levels of the APA were designated as APD_20_, APD_50_ and APD_90_, respectively. A rapid ventricular pacing (RVP) protocol, which involved pacing at a rate of 20 Hz for 1 s, was conducted with or without isoproterenol (ISO) challenge (1 μM) [22, 23] to induce triggered electrical activity or burst firing under 2 Hz pacing [22, 23]. Triggered activity was defined as spontaneous APs occurring without electrical stimuli, while burst firing referred to an accelerated rate of spontaneous APs. The frequency and duration of burst firing during the ISO challenge were recorded. RVOT tissues were evaluated both with and without the ISO challenge, followed by treatment with TP‐10 (10 μM) to assess its effects. To explore the signalling pathway associated with TP‐10 treatment, thapsigargin (2.5 μM, a sarcoendoplasmic reticulum calcium ATPase (SERCA) inhibitor) [24, 25, 26] and KT5823 (1 μM, a PKG inhibitor) [27] were administered. Thapsigargin inhibits the SERCA, leading to an increase in intracellular calcium levels. Using thapsigargin can help elucidate how the level of calcium cycling is modulated, particularly in relation to the cGMP pathway that may be influenced by TP‐10, thereby affecting cellular responses and electrophysiological properties.

### 2.2 Cardiomyocyte Isolation

Ventricular myocytes were isolated enzymatically using modified techniques based on established protocols [28]. In summary, hearts were cannulated via the aorta for Langendorff perfusion. Initially, the hearts were perfused with a normal Tyrode's solution for a duration of 10 min. Following this, the hearts underwent digestion with a calcium‐free enzyme solution containing 300 units/mL of collagenase (Type I; Sigma‐Aldrich) and 0.25 units/mL of proteinase (Type XIV; Sigma‐Aldrich) for 8–12 min. After perfusion, the hearts were removed from the cannulas, and the RVOTs were excised and dissected into smaller fragments. These fragments were subsequently triturated gently using a plastic transfer pipette in 50 mL of calcium‐free solution and filtered through a nylon mesh to yield single cardiomyocytes. The dissociated cells were then gradually transferred to the initial NaCl solution. The cardiomyocytes were maintained in this solution at 20°C–22°C and allowed to stabilise for a minimum of 30 min prior to experimentation. Only rod‐shaped cells displaying distinct striations and lacking granulation were utilised within 6–8 h for all experimental procedures. To evaluate the effects of TP‐10, the cells were treated with TP‐10 (10 μM) for monitoring intracellular calcium levels and conducting electrophysiological assessments. The normal Tyrode's solution consisted of 137 mM NaCl, 5.4 mM KCl, 1.8 mM CaCl_2_, 0.5 mM MgCl_2_, 10 mM glucose and 10 mM 4‐(2‐hydroxyethyl) piperazine‐1‐ethanesulfonic acid (HEPES), with pH maintained at 7.4 using NaOH. The calcium‐free solution contained 120 mM NaCl, 5.4 mM KCl, 1.2 mM KH2PO_4_, 1.2 mM MgSO_4_, 10 mM glucose, 10 mM HEPES and 10 mM taurine, also adjusted to a pH of 7.4 with NaOH.

### 2.3 Intracellular Ca^2+^ Monitoring

RVOT cardiomyocytes were stained with a calcium dye (10 μM Fluo‐3 AM) at 20°C–22°C for 30 min, and imaging was conducted as documented method [29, 30]. Fluorescence microscopy was executed using an inverted laser‐scanning confocal microscope (Zeiss LSM 510; Carl Zeiss, Jena, Germany). To address variations in dye concentrations, fluorescent signals were normalised by comparing the observed fluorescence (denoted as F) to the baseline fluorescence (F0). This normalisation, expressed as (F–F0)/F0, facilitated an accurate evaluation of transient changes in intracellular calcium concentration ([Ca^2+^]i) relative to baseline levels, effectively mitigating discrepancies in fluorescence intensity due to differing volumes of injected dye. Calcium transients were recorded using a stimulation frequency of 2 Hz. The sarcoplasmic reticulum (SR) calcium stores were assessed by rapidly administering 20 mM caffeine following a pulse stimulation train at 2 Hz for 30 s, with peak amplitudes of the caffeine‐induced calcium transient used for estimation. The SR calcium content was evaluated by integrating the inward sodium‐calcium exchanger (NCX) current triggered by the rapid application of 20 mM caffeine to voltage‐clamped cells, as previously described [30]. Initially, cells were held at −40 mV while being superfused with Tyrode's solution. Prior to the application of caffeine, an SR Ca^2+^ loading protocol was implemented, which involved a train of AP clamps at 1 Hz for 30 s. Following this loading phase, stimulation was halted, and the superfusate was swiftly replaced with an NT solution containing 20 mM caffeine. The Ca^2+^ released from the SR is primarily extruded from the cell via the NCX, resulting in an inward current. Consequently, the SR Ca^2+^ content can be quantified by integrating the inward NCX current [30].

### 2.4 Electrophysiological Measurement

#### 2.4.1 I_to_, I_Ca,L_, NCX current and I_Na,L_

The electrophysiological characteristics of RVOT cardiomyocytes were assessed using whole‐cell patch‐clamp techniques in the configuration with a patch clamp amplifier (Axopatch 1D, Axon Instruments, USA), as previously detailed in reference [31]. At the beginning of each experiment, a brief hyperpolarising pulse was delivered, shifting the holding potential from −50 to −55 mV for 80 ms. The resulting area under the capacitive current was divided by the applied voltage step, allowing for the determination of cell capacitance. Additionally, the series resistance was electronically compensated, reaching a compensation level of 60%–80%.

The transient outward current (Ito) was examined according to an established protocol [32]. Briefly, a 30‐ms pre‐pulse was delivered, shifting voltage from −80 mV to −40 mV to inactivate the Na^+^ channels, followed by a 300‐ms test pulse to a voltage of +60 mV in 10‐mV increments. CdCl_2_ (200 μM) was supplemented into the bath solution to inhibit I_Ca,L_. Ito was measured as the difference between the peak outward current and the steady‐state current. The I_Ca,L_ was measured as inward currents under voltage‐clamp with steps from a holding voltage of ‐50 mV to test voltages from −40 to +60 mV in 10‐mV increments for 300 ms [33]. The NCX current was examined using voltage‐clamp with test potentials between −100 and +100 mV from a holding potential of −40 mV in 20 mV increments for 300 ms. The amplitudes of NCX current were measured as Nickel‐sensitive currents as described in the method [33]. To measure the I_Na,L_, a step/ramp protocol, starting with a holding voltage of −100 mV, was followed by a test voltage to +20 mV for 100 ms and ramping back to a test voltage of −100 mV for 100 ms. The I_Na,L_ was measured as the tetrodotoxin‐sensitive current acquired when the voltage was ramped back to −100 mV test voltage as per the previously established protocol [33].

### 2.5 Western Blot Analysis

Western blotting was conducted to quantify calcium regulatory proteins, including SERCA2a, calcium/calmodulin‐dependent protein kinase II (CaMKII), phosphorylated CaMKII (pCaMKII), phospholamban (PLB) and phosphorylated PLB at Ser16 and Thr17. Ventricular cardiomyocytes were used to prepare whole‐cell lysates. The protein extraction and gel electrophoresis were conducted according to a previously established protocol [34]. The membranes were treated with the following primary antibodies: CaMKII (GTX111410; GeneTax; 1:2000), pCaMKII (ab32678; Abcam, Cambridge, UK; 1:2000), SERCA2a (sc‐376,235; Santa Cruz Biotechnology, Dallas, TX, USA; 1:1000), PLB (MA3‐922; Thermo Fisher Scientific; 1:5000), phosphorylated PLB at Ser16 (A010‐12;; 1:2000) or Thr17 (A010‐13AP; 1:5000), and GAPDH (M171‐7; MBL; 1:5000). Following this, the membranes were incubated with secondary IgG antibodies specific to either mouse (sc‐2056; Santa Cruz Biotechnology; 1:5000) or rabbit (sc‐2004; Santa Cruz Biotechnology; 1:5000). Immunoreactive proteins were visualised using enhanced chemiluminescence (GE Healthcare, Chicago, IL, USA) and quantified with ImageJ (National Institutes of Health, USA).

### 2.6 Data Analysis

Student's *t*‐test and Pearson's chi‐square test, conducted using SigmaPlot version 12 (Systat Software, San Jose, CA, USA), were utilised to analyse differences between the control and treatment groups. In this setting, ‘*n*’ represents the total number of cells derived from the total number of hearts (*n* = cells/hearts), whereas ‘*N*’ represents the number of animals used. Statistical significance was denoted by *, ** and *** for *p*‐values less than 0.05, 0.01 and 0.005, respectively.

## 3 Results

### 3.1 Electrical Activity in the Apex and Outlet Tissues of the Right Ventricle

**FIGURE 2:**
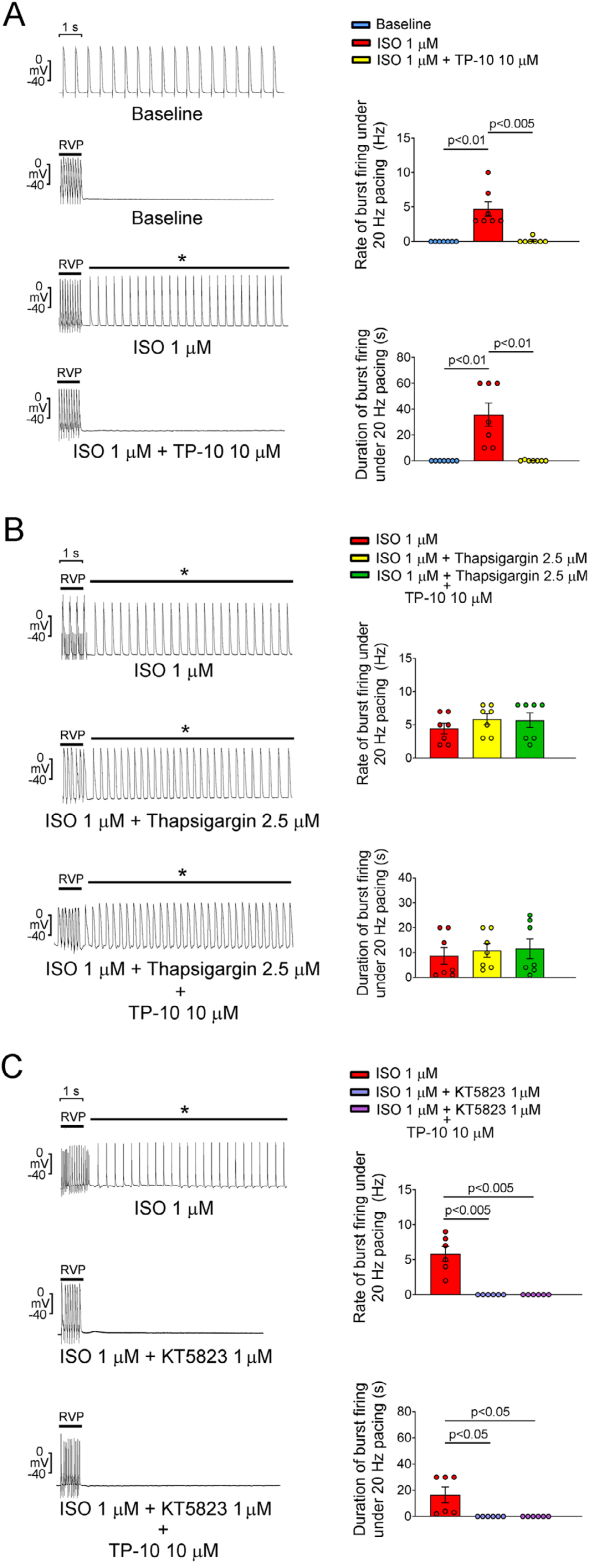
Effects of TP‐10, thapsigargin/TP‐10 or KT5823/TP‐10 cotreatment on ISO challenge‐induced bursting firing in RVOT tissues. (A) (Left) Representative recordings of post‐RVP electric activity in RVOT tissues at baseline, as well as treated with ISO and ISO/TP‐10. (Right) Mean rate and duration of post‐RVP bursting firing at baseline, under ISO treatment, and under ISO/TP‐10 cotreatment (*N* = 7). (B) (Left) Representative recordings of post‐RVP electric activity in ISO‐challenged, ISO/thapsigargin‐cotreated and ISO/thapsigargin/TP‐10‐cotreated RVOT tissues. (Right) Mean rate and duration of post‐RVP bursting firing in ISO‐challenged, ISO/thapsigargin‐cotreated and ISO/thapsigargin/TP‐10‐cotreated RVOT tissues (*N* = 7). (C) (Left) Representative recordings of post‐RVP electric activity in ISO‐challenged, ISO/KT5823‐cotreated and ISO/KT5823/TP‐10‐cotreated RVOT tissues. (Right) Mean rate and duration of post‐RVP bursting firing in ISO‐challenged, ISO/KT5823‐cotreated and ISO/KT5823/TP‐10‐cotreated RVOT tissues (*N* = 6). ISO, isoproterenol; RVOT, right ventricular outflow tract; RVP, rapid ventricular pacing.

RVOT exhibits distinctive electrophysiological characteristics compared to RAV [20]. These differences may contribute to the high arrhythmogenicity of the RVOT when exposed to pharmacological interventions or pathological stimuli. Therefore, we first tested the effect of TP‐10 on both the RAV and the RVOT. TP‐10 (10 μM) did not affect the RMP, APA, APD and contractile force in the RAV tissues (Figure 1). However, TP‐10 (10 μM) shortened the APD_20_, APD_50_ and APD_90_ in RVOT tissues (Figure 1). Furthermore, RVOT tissues challenged with ISO exhibited increased rates and prolonged durations of post‐RVP sustained burst firing compared to control ventricles, an effect that was eliminated following TP‐10 treatment at 10 μM (Figure 2A). To explore this protective mechanism of TP‐10, thapsigargin, an SERCA inhibitor, and KT5823, a PKG inhibitor, were used. After treatment with thapsigargin, TP‐10 did not suppress the post‐RVP bursting firing induced by ISO challenge in RVOT tissues (Figure 2B). Both KT5823 treatment and KT5823/TP‐10 cotreatment abolished the post‐RVP bursting firing induced by ISO challenge in RVOT tissues (Figure 2C).

### 3.2 Amplitudes of Calcium Transient and SR Calcium Content

**FIGURE 3:**
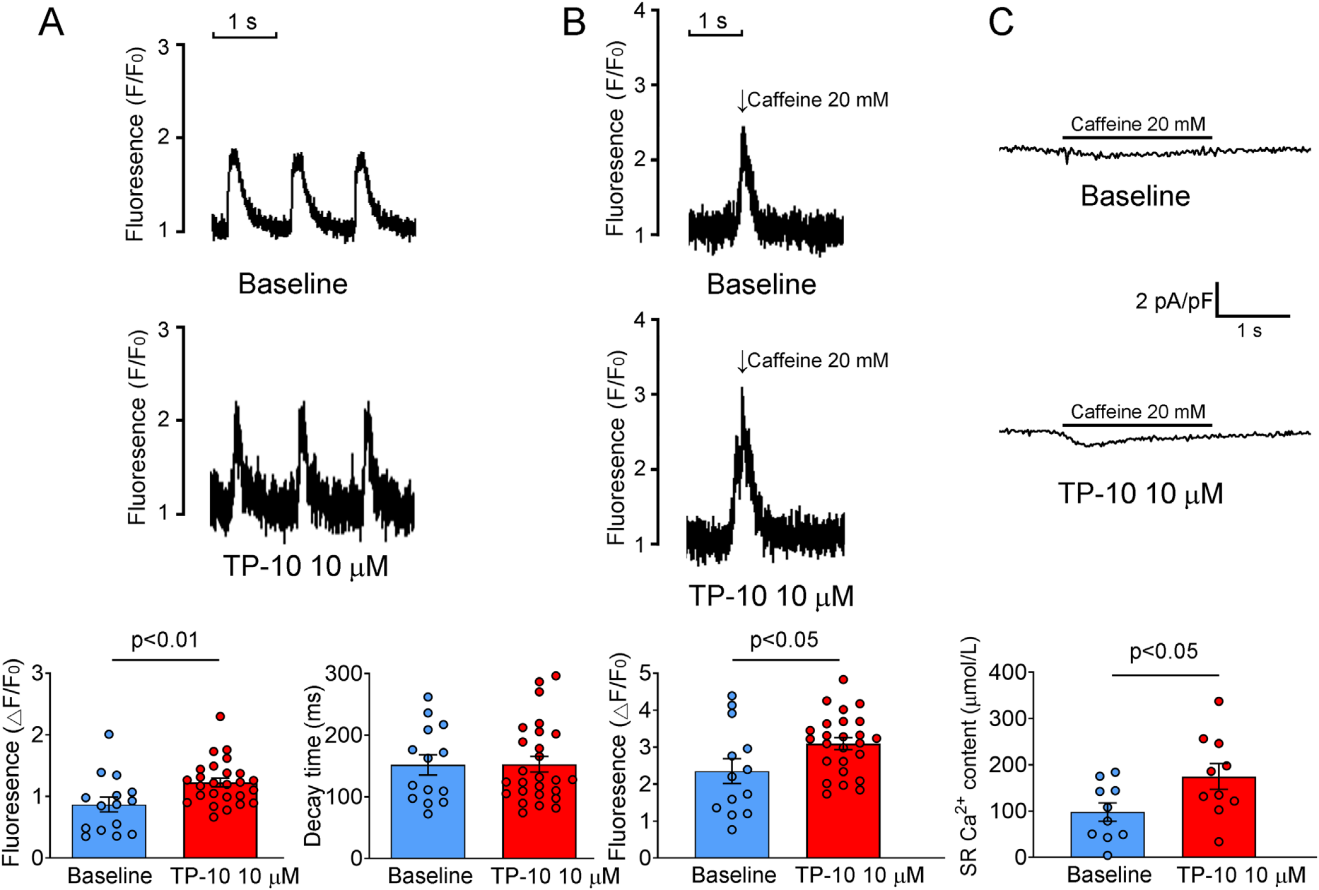
Effect of TP‐10 on Ca^2+^ transient and SR Ca^2+^ content. (A) (Upper and middle) Typical traces of steady‐state Ca^2+^ transient in RVOT cardiomyocytes treated with TP‐10 and baseline control (without TP‐10), as well as (lower) the mean values (baseline group *n* = 15/4, *N* = 4; TP‐10 group *n* = 26/5, *N* = 4). (B) (Upper and middle) Typical traces of caffeine‐elicited Ca^2+^ transient in RVOT cardiomyocytes treated with TP‐10 and baseline control (without TP‐10), as well as (lower) the mean values (baseline group *n* = 13/4, *N* = 4; TP‐10 group *n* = 24/5, *N* = 5). (C) (Upper and middle) Typical recording of SR Ca^2+^ content determined by integral of inward NCX current induced by fast caffeine application in voltage‐clamped RVOT cardiomyocytes treated with TP‐10 and baseline control (without TP‐10), as well as (lower) the mean values (baseline group *n* = 10/5, *N* = 5; TP‐10 group *n* = 10/5, *N* = 5). NCX, sodium‐calcium exchanger; RVOT, right ventricular outflow tract; SR, sarcoendoplasmic reticulum.

The amplitudes of steady‐state Ca^2+^ transients were higher in TP‐10‐treated cardiomyocytes compared to the controls (Figure 3A). Additionally, the caffeine‐induced Ca^2+^ transients were also elevated in TP‐10‐treated cardiomyocytes relative to the controls (Figure 3B). The SR Ca^2+^ content, determined by the integral of the inward NCX current under voltage‐clamp conditions, was increased in TP‐10‐treated cardiomyocytes compared to those in the controls (Figure 3C).

### 3.3 I_to_, I_Ca_ _,L_, NCX Current and I_Na_ _,L_

**FIGURE 4:**
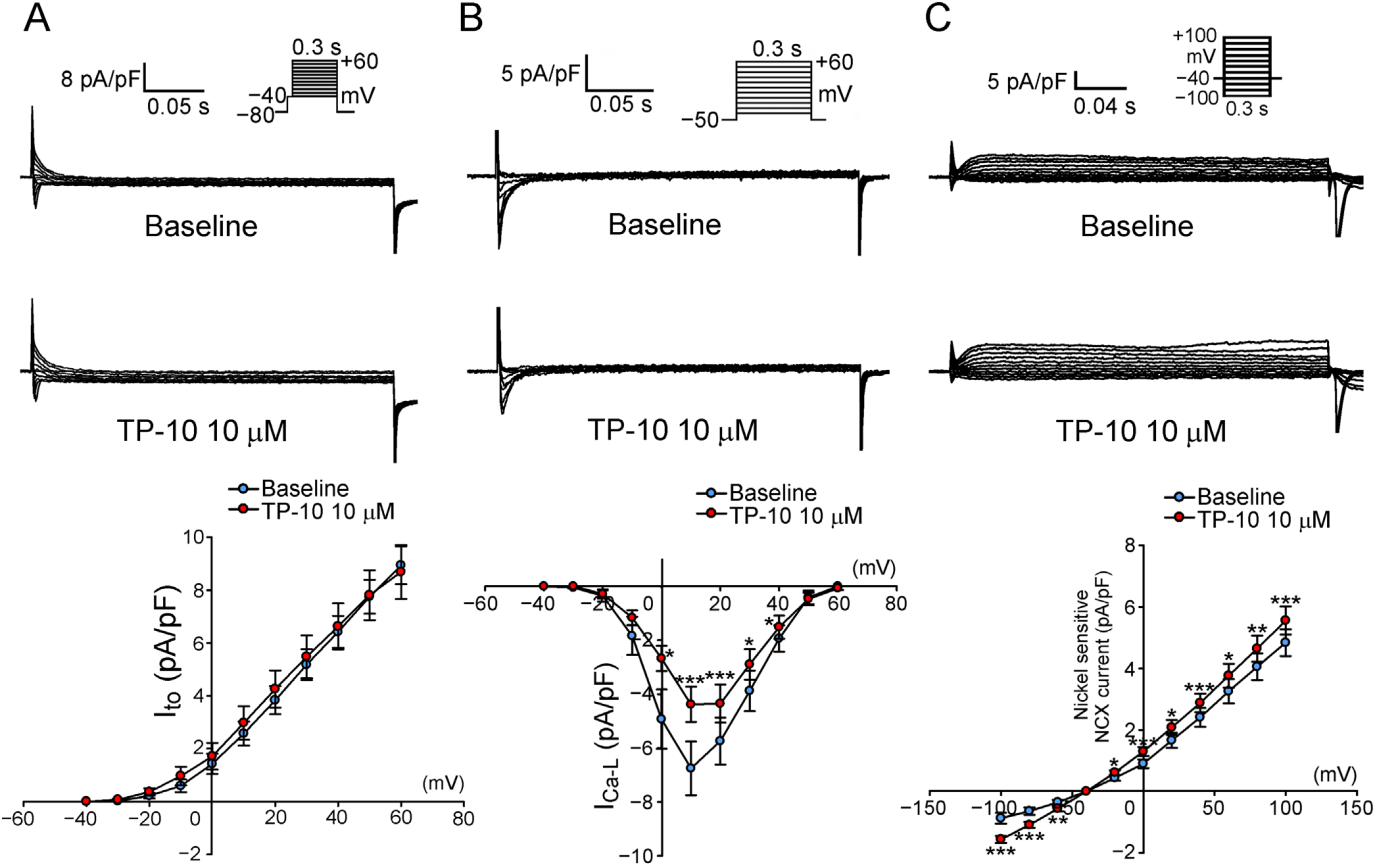
Effect of TP‐10 on I_to_, I_Ca,L_ and NCX current. (A) (Upper and middle) Representative traces of I_to_ in RVOT cardiomyocytes treated with TP‐10 and baseline control (without TP‐10), as well as (lower) the I–V relationship (*n* = 8/4, *N* = 4). (B) (Upper and middle) Representative traces of I_Ca,L_ in RVOT cardiomyocytes treated with TP‐10 and baseline control (without TP‐10) as well as (lower) the I–V relationship (*n* = 9/3; *p < 0.05; ***p < 0.005) (C) (Upper and middle) Representative traces of NCX current in RVOT cardiomyocytes treated with TP‐10 and baseline control (without TP‐10), as well as (lower) the I–V relationship (*n* = 10/4, *N* = 4; *p < 0.05; ***p < 0.005). I–V, current–voltage; NCX, sodium‐calcium exchanger; RVOT, right ventricular outflow tract.

**FIGURE 5:**
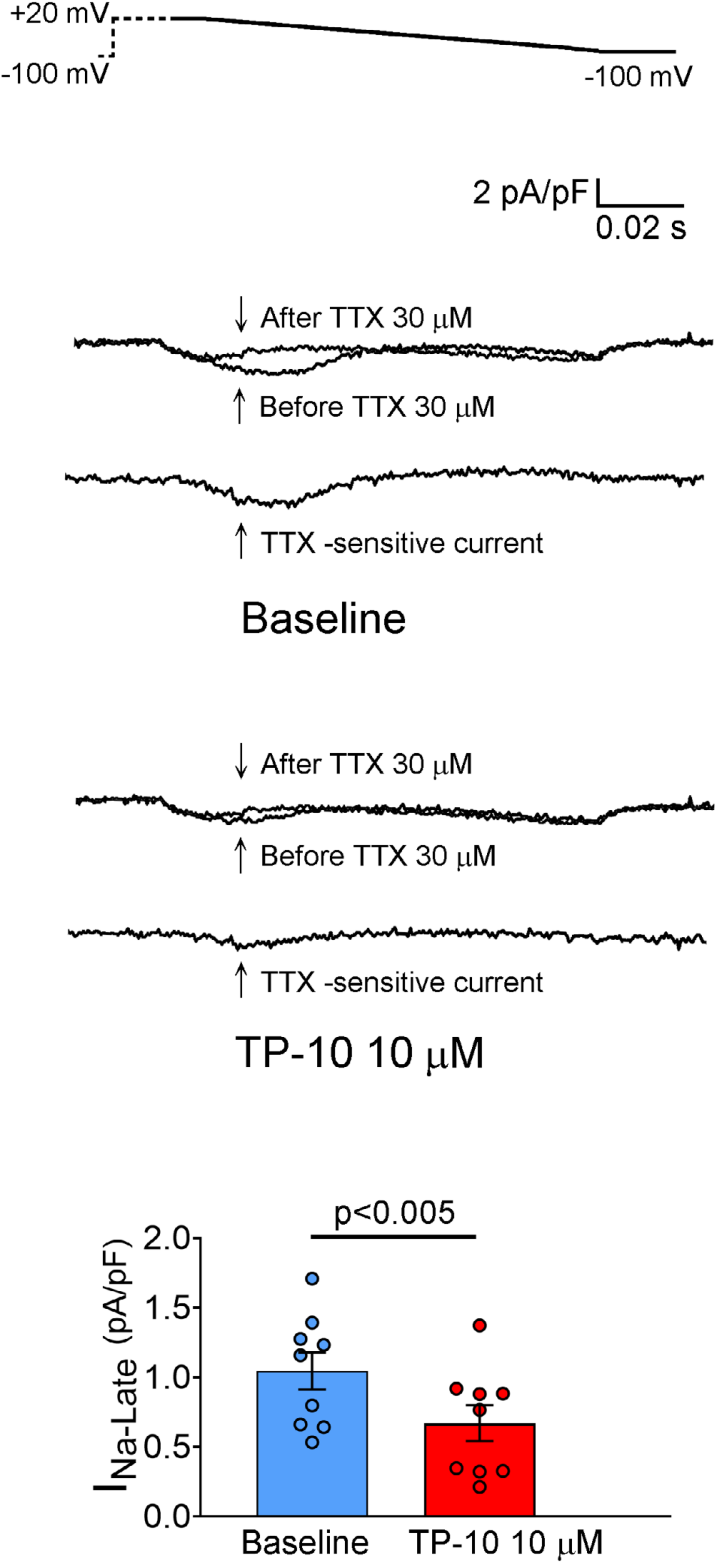
Effect of TP‐10 on I_Na,L_. (Left) Representative tracings of I_Na,L_ (TTX‐sensitive current) with the voltage‐clamp protocol shown in the inset above the current traces, and (Right) the mean values of RVOT cardiomyocytes treated with TP‐10 and baseline control (without TP‐10) (baseline group *n* = 9/3, *N* = 3; TP‐10 group *n* = 9/3, *N* = 3;pA/pF to pA/pV, respectively). RVOT, right ventricular outflow tract; TTX, tetrodotoxin.

There was no difference in I_to_ between RVOT cardiomyocytes treated with TP‐10 and controls (Figure 4A). The peak I_Ca,L_ was smaller in TP‐10‐treated RVOT cardiomyocytes than in controls (Figure 4B). However, the NCX current was higher in TP‐10‐treated RVOT cardiomyocytes compared to controls (Figure 4C). Meanwhile, TP‐10‐treated RVOT cardiomyocytes exhibited a reduced density of I_Na,L_ compared to controls (1.05 ± 0.13 vs. 0.67 ± 0.13 pA/pF; *p* < 0.005) (Figure 5).

### 3.4 Levels of Ca^2+^ Regulatory Proteins

**FIGURE 6:**
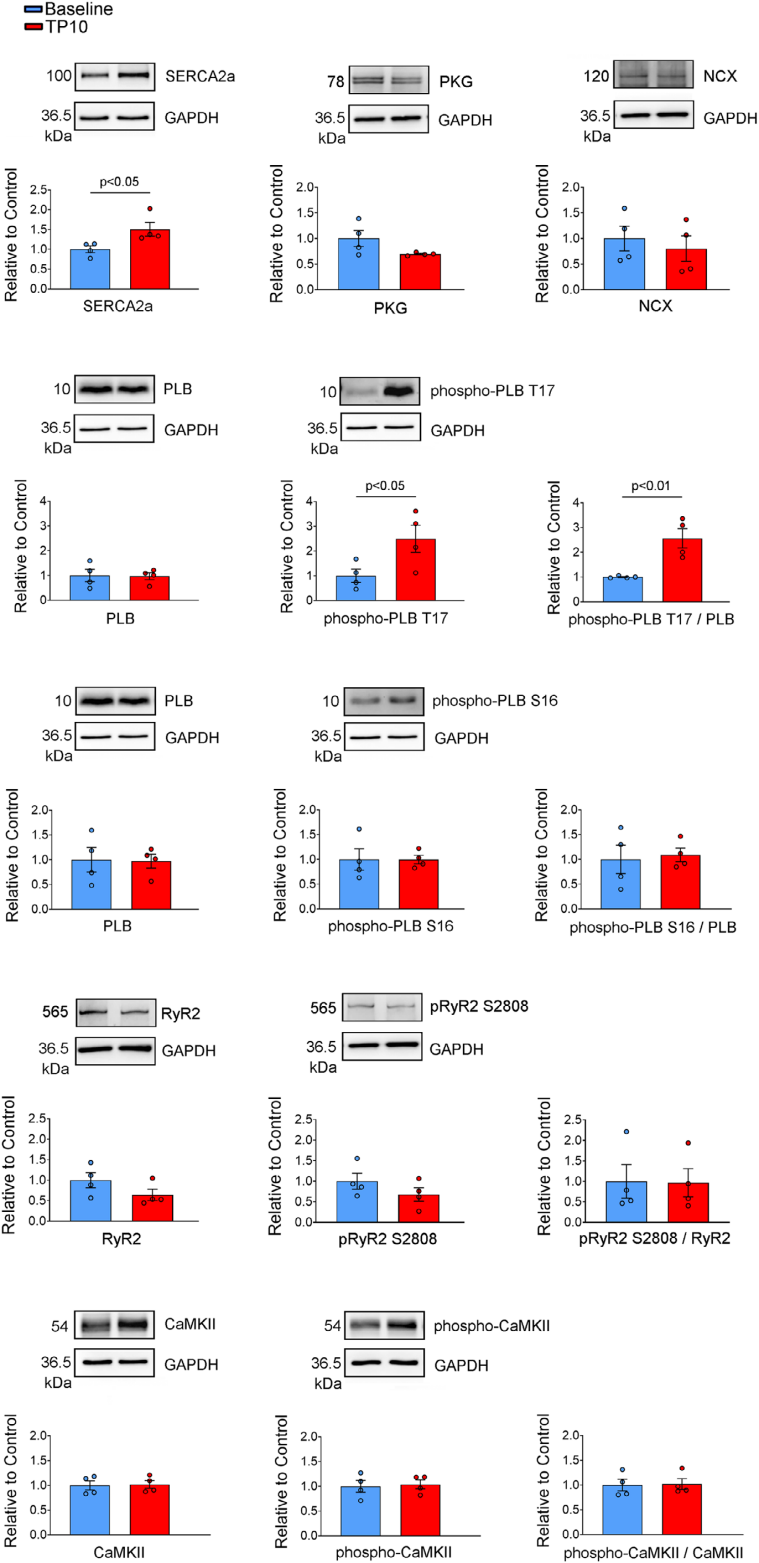
Quantification of Ca^2+^ regulatory proteins in control and TP‐10‐treated RVOT cardiomyocytes. Representative immunoblot and normalised densitometric protein levels of phosphorylated CaMKII, CaMKII, SERCA2a, PLB, PLB pSer16 and PLB pThr17 in RVOT cardiomyocytes treated with TP‐10 and baseline control (without TP‐10). GAPDH was used as an internal control (baseline group *N* = 4, TP‐10 group *N* = 4). We used the same sample from the same animal in the same gel for PLB, phospho‐PLB S16, phospho‐PLB T17 and GAPDH western blotting. Consequently, the representative GAPDH is the same for PLB, phospho‐PLB S16 and phospho‐PLB T17. Additionally, the PLB/GAPDH values used for calculating the ratio of phosphorylated PLB at S16 or T17 are identical. PLB, phospholamban; CaMKII, Ca(2+)/calmodulin‐dependent protein kinase II; RVOT, right ventricular outflow tract; SERCA2a, sarcoendoplasmic reticulum calcium ATPase 2a.

The protein levels of pCaMKII and CaMKII did not differ between TP‐10‐treated RVOT cardiomyocytes and the controls (Figure 6). TP‐10‐treated RVOT cardiomyocytes had higher protein levels of SERCA2a than the controls (Figure 6). While the expression of PLB and PLB pSer16 protein did not change in TP‐10‐treated RVOT cardiomyocytes compared to the controls, the expression of PLB pThr17 increased in TP‐10‐treated RVOT cardiomyocytes (Figure 6).

## 4 Discussion

The current study demonstrates that TP‐10 mitigated ISO challenge‐induced ventricular arrhythmia in the RVOT. Specifically, TP‐10 treatment suppressed both the rates and durations of burst firing in ISO‐challenged RVOT tissues. This suppressive effect was nullified when co‐treated with thapsigargin but remained evident with co‐treatment of KT5823. Thapsigargin inhibits the SERCA, resulting in an increase in intracellular calcium concentrations. In the present study, the suppressive effect of TP‐10 on isoproterenol‐induced burst firing in RVOT tissues was negated when co‐treated with thapsigargin. This finding suggests that TP‐10's mechanism involves the modulation of calcium handling influenced by SERCA activity, indicating a close relationship between TP‐10's effects and calcium regulation. KT5823, a PKG inhibitor, also demonstrated that co‐treatment suppressed burst firing, implying that TP‐10 may operate through PKG‐independent cGMP‐mediated pathways. Specifically, PKG inhibition may diminish the effects of isoproterenol stimulation on ICa‐L and the spontaneous activity of cardiac tissues by modulating cAMP levels and PKA activity [35].

**FIGURE 7:**
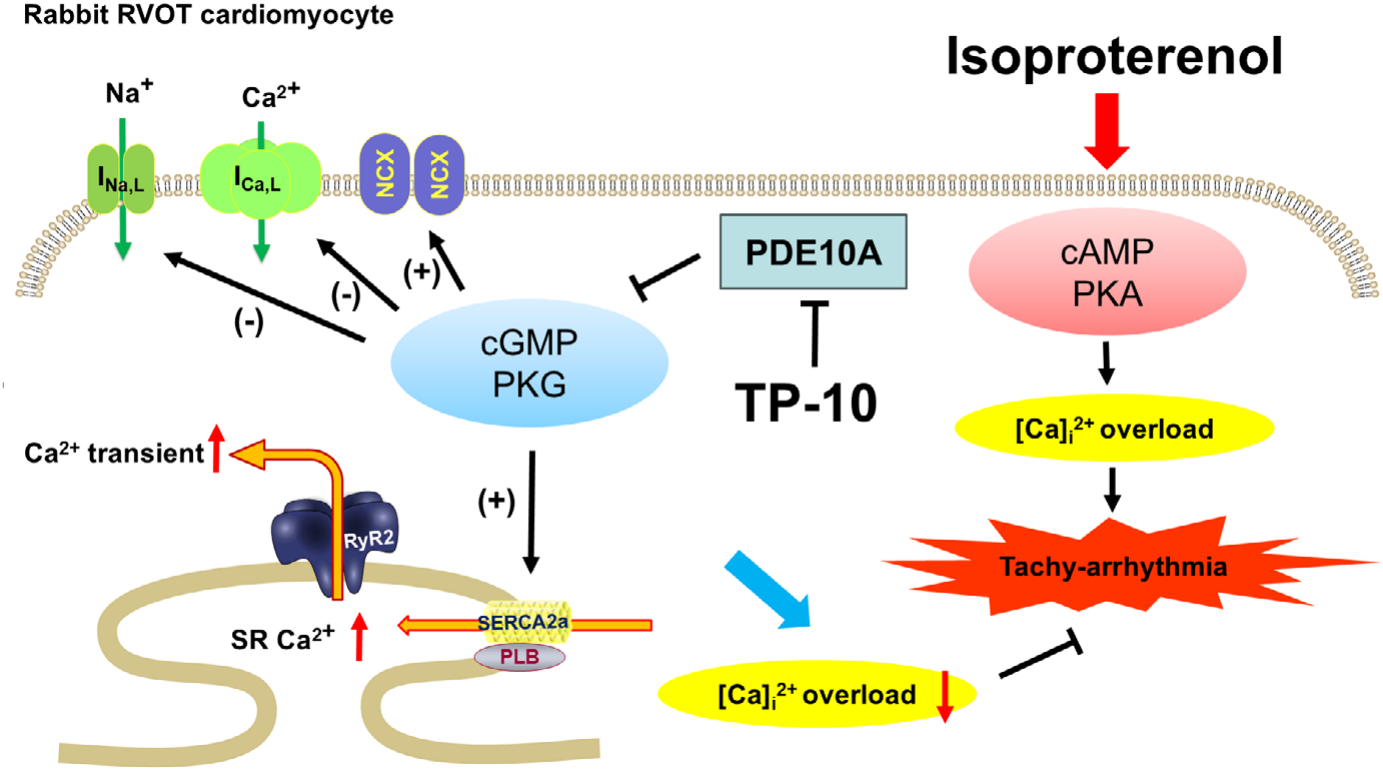
Proposed anti‐arrhythmia mechanism of TP‐10. TP‐10 enhances the cGMP/PKG pathway, resulting in reduced L‐type Ca^2+^ current and late Na^+^ current, as well as increased NCX current and upregulated SERCA2a function. These effects collectively increase SR Ca^2+^ content and Ca^2+^ transients, thereby attenuating isoproterenol‐induced intracellular Ca^2+^ overload that promotes tachyarrhythmia. cAMP, cyclic adenosine monophosphate; cGMP, cyclic guanosine monophosphate; NCX, sodium‐calcium exchanger; PDE10A, phosphodiesterase 10A; PKA, protein Kinase A; PKG, protein kinase G; PLB, phospholamban; RVOT, right ventricular outflow tract; RyR2, ryanodine receptor 2; SERCA2a, sarcoplasmic reticulum calcium ATPase 2a; SR, sarcoplasmic reticulum.

Additionally, TP‐10 enhanced Ca^2+^ transients and SR Ca^2+^ content in RVOT cardiomyocytes. While TP‐10 reduced L‐type Ca^2+^ currents, it increased NCX currents. Furthermore, TP‐10 decreased late Na^+^ currents and enhanced the function of SERCA. These findings suggest that TP‐10 modulates Ca^2+^ handling, potentially through cyclic guanosine monophosphate (cGMP) signalling, thereby preventing arrhythmic events in ISO‐challenged RVOT. The proposed anti‐arrhythmic mechanism of TP‐10 is illustrated in Figure 7.

Previous studies have demonstrated that PDE inhibition can augment Ca^2+^ transients, which aligns with our findings of the present study. In addition, we propose that observed increases in Ca^2+^ transients and SR Ca^2+^ stores may be attributed to the upregulation of SERCA2a induced by TP‐10. Although PDE inhibitors are known to augment cAMP levels, leading to PKA‐mediated enhancement of L‐type Ca^2+^ current and an inotropic effect, our data indicate that the inhibition of PDE10A with TP‐10 actually reduced L‐type Ca^2+^ current. This suggests that the inotropic mechanism of TP‐10 may not operate through the cAMP pathway but rather through the cGMP pathway. This hypothesis is supported by our data indicating that the inhibition of TP‐10 and PKG effectively abolished arrhythmias induced by ISO challenge. This suggests that targeting these pathways may be crucial for managing arrhythmias in this context.

Our data show that ISO challenge induced burst firing in RVOT tissues, which is suggested to be attributed to intracellular Ca^2+^ overload caused by the activated β‐adrenergic‐cAMP‐PKA pathway, leading to an increased probability of ectopic electrical activity, such as delayed after depolarisations [36]. TP‐10 suppressed ISO challenge‐induced burst firing in RVOT tissues. We propose that this effect is mediated through the cGMP/PKG signalling pathway, which reduces Ca^2+^ influx via L‐type Ca^2+^ channels [37]. Our results also show shortened APD in TP‐10‐treated RVOT ventricles. Shortening of the AP reduces Ca^2+^ influx, resulting in decreased intracellular Ca^2+^ load. In fact, the cGMP/PKG signalling pathway is proposed to provide cardioprotection by modulating Ca^2+^ homeostasis and reducing the production of reactive oxygen species [38].

The activity of SERCA is modulated by PLB. When PLB is unphosphorylated, it inhibits SERCA by reducing the ATPase's affinity for calcium. However, when PLB is phosphorylated, this inhibition is lifted, leading to an increase in SERCA activity. PLB has two key phosphorylation sites: Ser16, which is phosphorylated by cAMP‐dependent protein kinase, and Thr17, which is targeted by CaMKII [39, 40]. While the role of cGMP signalling in regulating SERCA activity and cardiomyocyte function is not well understood, our data indicate that TP‐10 increases phosphorylated PLB at Thr17, likely through cGMP/PKG signalling. The upregulation of SERCA leads to increased SR Ca^2+^ content and enhanced Ca^2+^ transients.

Research indicates that TP‐10 possesses cardioprotective effects. Chen et al. demonstrated that the PDE10A inhibitor TP‐10 elevates cAMP and cGMP levels in cardiac myocytes and fibroblasts, thereby mitigating pathological hypertrophy induced by angiotensin II, phenylephrine and isoproterenol [9]. The study proposes that PDE10A inhibition may represent a promising therapeutic approach for heart failure. However, the specific mechanism of PDE10A‐mediated cAMP and cGMP signalling in the modulation of hypertrophy and fibrosis remains unclear. Our study investigates the effects of TP‐10 on calcium handling in the isoproterenol‐challenged RVOT, emphasising its influence on intracellular calcium dynamics via cGMP pathways. Our data suggest that TP‐10‐mediated cGMP signalling regulates calcium handling and reduces the risk of arrhythmias in the challenged RVOT. Inhibition of PDE10A may enhance cardiac structure and function, positioning it as a potential target for the treatment of heart failure and arrhythmias.

The study's limitations include a small sample size of New Zealand white rabbits, which may affect the generalisability of the findings to humans. The use of isolated cardiomyocytes may not fully capture the complex interactions present in vivo, and the focus on specific cGMP‐mediated pathways leaves other potential factors influencing arrhythmogenesis unexplored. In addition, the research primarily addressed ISO‐induced arrhythmias, suggesting a need for exploration of other arrhythmic mechanisms.

In conclusion, TP‐10 mitigates ISO‐induced ventricular tachyarrhythmia in the rabbit RVOT. Our findings indicate that TP‐10 modulates intracellular Ca^2+^ regulation in cardiomyocytes through the cGMP pathway, thereby providing antiarrhythmic effects.

## Author Contributions

**Feng‐Zhi Lin:** data curation (equal), formal analysis (equal), investigation (equal). **Yao‐Chang Chen:** investigation (equal), methodology (equal), writing – original draft (equal). **Hsiang‐Yu Yang:** funding acquisition (equal), supervision (equal), writing – original draft (equal), writing – review and editing (equal). **Wei‐Shiang Lin:** supervision (equal), validation (equal), writing – original draft (equal). **Yen‐Yu Lu:** formal analysis (equal), investigation (equal), writing – original draft (equal).

## Conflicts of Interest

The authors declare no conflicts of interest.

## Supporting information


**Figure S1.** The echocardiography data of rabbits. The mean heart rate, EF %, LV end systolic internal dimension and LV end diastolic dimension of the rabbits are 272.0 ± 12.0 (beats/min), 66.7 ± 3.8 (%), 8.8 ± 0.9 (mm) and 13.3 ± 1.0 (mm), respectively.

## Data Availability

The data supporting the findings of this study can be requested from the corresponding author upon reasonable inquiry.
