# Supplementary figures and images for: Phosphodiesterase 10A Inhibitor Modulates Right Ventricular Outflow Tract Electrophysiological Activities and Calcium Homeostasis via the cGMP/PKG Pathway
Source: J Cell Mol Med. 2025 Mar 11;29(5):e70480. doi: 10.1111/jcmm.70480 (PMC11897058; doi:10.1111/jcmm.70480)

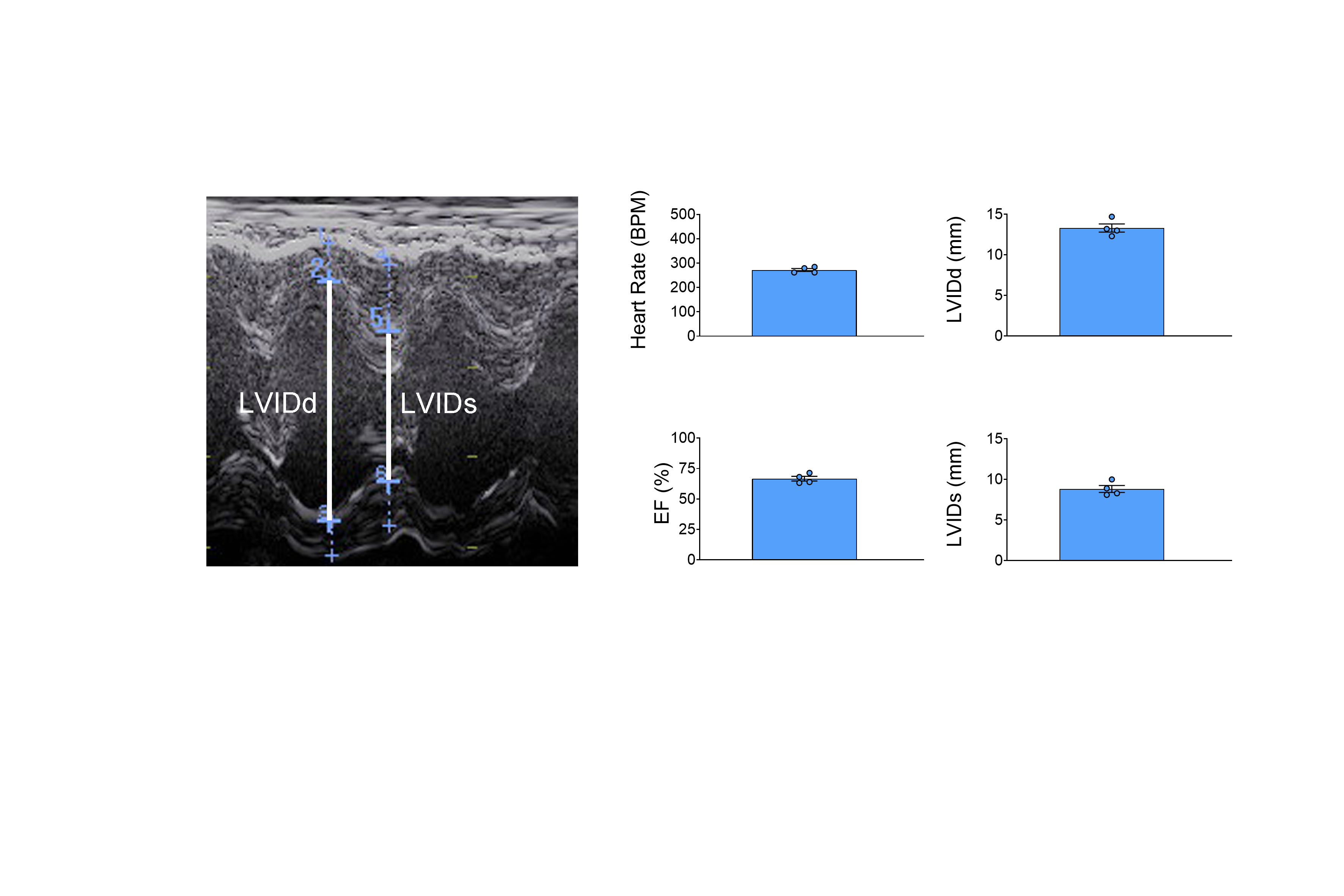

Supplement: Supplementary file 1 — Figure S1. The echocardiography data of rabbits. The mean heart rate, EF %, LV end systolic internal dimension and LV end diastolic dimension of the rabbits are 272.0 ± 12.0 (beats/min), 66.7 ± 3.8 (%), 8.8 ± 0.9 (mm) and 13.3 ± 1.0 (mm), respectively. [file JCMM-29-e70480-s001.tif]
